# The contribution of differences in adiposity to educational disparities in mortality in the United States

**DOI:** 10.4054/DemRes.2017.37.54

**Published:** 2017-12-07

**Authors:** Yana C. Vierboom

**Affiliations:** 1 Population Studies Center, University of Pennsylvania, Philadelphia, USA

## Abstract

**BACKGROUND:**

There are large differences in life expectancy by educational attainment in the United States. Previous research has found obesity’s contribution to these differences to be small. Those findings may be sensitive to how obesity is estimated.

**METHODS:**

This analysis uses discrete-time logistic regressions with data from the National Health and Nutrition Examination Survey (NHANES), pooled from 1988 to 1994 and 1999 to 2010, to estimate the contribution of differences in adiposity, or body fat, to educational differences in mortality. I show that results depend upon the measure of adiposity used: body mass index (BMI) at the time of survey or lifetime maximum BMI.

**RESULTS:**

College graduates were less likely than high school graduates to be obese at the time of survey (25% vs. 34.6%, respectively) and were also less likely to have ever been obese (35.7% vs. 49.4%, respectively). Lifetime maximum BMI performed better than BMI at the time of survey in predicting mortality using criteria for model selection. Differences in maximum BMI were associated with between 10.3% and 12% of mortality differences between college graduates and all others, compared to between 3.3% and 4.6% for BMI at the time of survey. Among nonsmokers, between 18.4% and 27.6% of mortality differences between college graduates and all others were associated with differences in maximum BMI.

**CONTRIBUTION:**

Adiposity is an overlooked contributor to educational differences in mortality. Previous findings that obesity does not contribute to educational disparities were based on BMI at the time of survey, which is less informative than maximum BMI. The contribution of adiposity to educational mortality differences will likely grow as smoking prevalence declines. Health surveys should collect information on weight history.

## 1. Introduction

### 1.1 The socioeconomic gradient in mortality

Life expectancy and disease patterns follow a well-documented socioeconomic gradient in many high-income countries (see, e.g., Elo 2009; Hayward, Hummer, and Sasson 2015; Laditka and Laditka 2016; Mackenbach et al. 2008). Between 1999 and 2011, for example, life expectancy for a 40-year-old white woman with a high level of education in the United States was 42.7 years, compared to 29.6 years for a woman with a low level of education (Laditka and Laditka 2016). Substantial disparities in mortality rates by educational attainment have similarly been observed in many European countries (Mackenbach et al. 2008). Recent evidence suggests that differentials in the United States and elsewhere may be widening (see, e.g., Mackenbach et al. 2015; National Academies of Sciences, Engineering, and Medicine 2015), adding further urgency to an already urgent issue.

An extensive body of research seeks to identify the mechanisms driving the relationship between education and mortality. One branch of this work focuses on the impact of modifiable health behaviors, such as smoking, alcohol consumption, drug use, and obesity. Obesity, defined as having a body mass index (BMI) of 30 or greater, is a salient factor to consider in view of its growing prevalence in many countries (Devaux and Sassi 2013; Flegal et al. 2007a; Ogden et al. 2010, 2015; Robertson, Lobstein, and Knai 2007). This global trend is well illustrated in the United States (Global Health Observatory 2016; Finucane et al. 2011). In 1980, for example, 16% of US women and 12% of US men were considered obese, compared to 36% of women and 34% of men in 2014 (Global Health Observatory 2016). Though obesity rates have increased for all segments of the US population in recent decades, poorer and less educated people remain more likely to be obese (Chang and Lauderdale 2005; Ogden et al. 2010; Yu 2012), an association that may have strengthened during the Great Recession (Wang, Wang, and Halliday 2016). This relationship is worrisome, given the health and mortality risks associated with obesity. Stokes and Preston (2016b) estimate that nearly one in six deaths occurring in the United States between 1988 and 2004 can be attributed to obesity. Obesity influences health and mortality through a variety of channels, such as by increasing the risk of diabetes (Abdullah et al. 2011), cardiovascular diseases (Tarleton et al. 2014), cancer (Wolin, Carson, and Colditz 2010), and disability (Alley and Chang 2007; Reynolds, Saito, and Crimmins 2005). Growing educational disparities in obesity prevalence could exacerbate existing mortality differentials.

Despite the health implications of obesity and its possibly strengthening inverse relationship with socioeconomic status (SES), obesity has not been adequately examined as a determinant of socioeconomic mortality differentials in the United States. Surprisingly, research that has explored this topic, such as the work on health behaviors by Mehta, House, and Elliott (2015) or Montez and Zajacova (2013), finds no significant contribution of adiposity, or body fat, to socioeconomic differences in mortality. It is possible that these and other findings are sensitive to bias in estimating the mortality consequences of obesity, as outlined in the following section.

### 1.2 Maximum BMI: A better way to estimate adiposity in studies of mortality disparities

Prior studies on health behaviors and SES commonly estimate adiposity using one cross-sectional observation of BMI, a weight-to-height ratio calculated as weight in kilograms over height in meters squared, measured at the time of survey. BMI at the time of survey can be a problematic measure in quantifying the relationship between adiposity and mortality because it is susceptible to bias from reverse causation due to illness. Formerly-obese individuals who have lost weight from an illness may present a healthy BMI at survey time but have an elevated risk of mortality due to the underlying illness. This process is illustrated by the finding that mortality risks are often highest among individuals who have lost weight (Stokes and Preston 2016b; Yu et al. 2017). The process of ill individuals being selected out of the obese population artificially inflates mortality rates for lower BMI ranges and deflates mortality rates for higher ones (Stokes 2014; Stokes and Preston 2016a, 2016b). As a result, estimates of the risks associated with obesity may be biased downwards, sometimes creating the illusion of a survival advantage for overweight or obese individuals (Stokes and Preston 2016a). Since less-educated individuals are more likely to contract illnesses that result in weight loss, as I demonstrate in Section 3.1 below, reverse causation is of particular concern in studying socioeconomic mortality differentials.

One common approach to addressing reverse causation due to illness when using longitudinal data is to delay the onset of analysis, often by five years, to exclude individuals who are most ill (Flegal et al. 2007b; Global BMI Mortality Collaboration 2016). This approach is imperfect, as it reduces sample size and statistical power (Hu 2008; Stokes and Preston 2016a). Additionally, weight loss trajectories vary considerably by age and disease (Alley et al. 2010). A second method excludes individuals who report having a disease associated with weight loss (Flegal et al. 2007b; Global BMI Mortality Collaboration 2016). In addition to reducing sample size and missing undiagnosed cases, conditioning on diseases that may be on the pathway from obesity to mortality can underestimate the relative risks associated with obesity (Hu 2008).

One recently proposed solution for addressing reverse causation bias that avoids some of the pitfalls of other methods is to use lifetime maximum BMI, based on highest-ever weight (Stokes 2014; Stokes and Preston 2016a, 2016b). Because maximum weight is likely attained at a time free of illness, this approach minimizes the likelihood of illness-induced weight loss without reducing sample size, conditioning on prediagnosed morbidity, or otherwise restricting the generalizability of the findings (Stokes and Preston 2016a).

The advantages of maximum BMI are not limited to reducing bias from reverse causation. A growing literature documents that elements of weight history are predictive of mortality (Preston, Mehta, and Stokes 2013). The mortality risks of obesity, for example, increase with the duration of obesity (Abdullah et al. 2011, 2014). Given that duration and peak weight were positively correlated in one analysis at 0.62 (Mehta et al. 2014), a weight history measure like maximum BMI captures more information about factors influencing mortality than does a single, cross-sectional observation. This measure is especially salient for examining socioeconomic differentials in health and mortality, since we know from existing research that SES and health behaviors are closely linked. Pampel, Krueger, and Denney (2010) identify a set of broad mechanisms through which SES influences health behaviors, many of which predict socioeconomic differences in weight history. Individuals of lower SES, for example, tend to experience more negative life events which may trigger weight gain, may not have information about the risks of excess weight, and may lack access to resources that make it easier to maintain a healthy weight.

An early variant of lifetime maximum BMI, peak BMI, was first used in a cohort study of Finnish adults by Mehta et al. (2014). The authors calculated peak BMI from self-reported weight at ten-year age intervals, finding that peak BMI was positively associated with increased mortality, net of BMI at survey time. Recent research building on Mehta et al.’s work has consistently found an association between mortality and having ever been obese. In their comparison of maximum and survey time BMI in three large cohort studies, Yu et al. (2017) find that the power of having ever been obese to predict mortality is far greater than the predictive power of obesity at the time of survey. Research by Stokes and Preston (2016b) uses model selection criteria to conclude that maximum BMI is a more robust predictor of mortality than the survey time BMI. In one of the first studies to apply the measure beyond testing its robustness, Elo, Mehta, and Preston (2017) use it to examine racial differences in mortality in the United States, finding that differences in maximum BMI account for 29% of black–white differences in mortality among women and 1% among men.

Although peak BMI was initially developed using cohort data, many studies, including the present one, calculate maximum BMI in cross-sectional surveys using measured height and self-reported maximum weight (Elo, Mehta, and Preston 2017; Stokes and Preston 2016a, 2016b). While bias introduced by using self-reported weight is a concern, Preston, Fishman, and Stokes (2015) demonstrate that biases are sharply reduced by using a continuous measure of BMI. Additionally, the biases may not be in the expected direction: while individuals generally underestimate current weight (Preston, Fishman, and Stokes 2015), they appear to overestimate past weight (Stokes and Ni 2016).

### 1.3 Study aim

This study investigates the contribution of adiposity to educational mortality differentials in the United States, using several measures of adiposity. Previous research finding no contribution of obesity to educational mortality differences relies on BMI at the time of survey, a measure which underestimates the risks of obesity (Stokes and Preston 2016a, 2016b). Maximum BMI has already been applied to investigate racial differences in mortality (Elo, Mehta, and Preston 2017), but has not before been used to examine educational differences in mortality. Accurately quantifying adiposity’s contribution to educational mortality differentials is crucial for reducing mortality disparities in a context of increasing obesity prevalence.

Although this analysis uses data from the United States, given the country’s vanguard position in a global trend of rising obesity prevalence, the findings of this analysis are likely generalizable to other high-income countries with comparable inequalities in mortality and obesity by educational attainment. Existing research indicates that many European countries may fit this description (Devaux and Sassi 2013; Mackenbach et al. 2008; Robertson, Lobstein, and Knai 2007).

## 2. Data and methods

### 2.1 Data source and sample

I use data from the National Health and Nutrition Examination Survey (NHANES), an annual cross-sectional health survey administered by the National Center for Health Statistics. I combine data from NHANES III (1988–1994) and NHANES Continuous waves (1999–2010), weighted to be nationally representative of the non-institutionalized US population. In addition to participating in detailed in-person interviews, adult respondents visit mobile examination centers for physical examinations. The participation rates for the years included in the sample range from 75% to 80% (Centers for Disease Control and Prevention 2015). Each wave has been linked to the National Death Index through December 2011, allowing for mortality follow-up. More detailed information on survey design and sampling procedures are available elsewhere (Centers for Disease Control and Prevention 1996; Johnson et al. 2013).

I restrict the study population for the main analysis to respondents aged 40 to 74 at the time of survey who were physically examined, not pregnant, and not missing information on height or weight measures. I exclude respondents missing information on educational attainment (n = 49) and smoking (n = 7). Since the aim of this analysis is to examine the contribution of obesity to mortality differences, I exclude respondents who have always been underweight (maximum BMI <18.5) (n = 18) or who are currently underweight (survey BMI <18.5) (n = 261). I also exclude respondents with maximum BMI values of 60 or greater (equivalent to being 5′10″ tall and weighing 420 pounds) (n = 70), to avoid the influence of outliers. Respondents are censored upon reaching age 85 during mortality follow-up. The final sample consists of 22,703 respondents experiencing 3,784 deaths from all causes across 215,066 person-years of follow-up. The mean length of follow-up is 10.9 years.

### 2.2 Variable design

#### 2.2.1 Outcome variable

The dependent variable is all-cause mortality as registered in the National Death Index between the time of participation in the survey and December 31, 2011.

#### 2.2.2 Education

Earlier NHANES waves (1988–1994) measured education as years of completed schooling, ranging from 0 to 17 years. Later waves (1999–2010) collected this information using a five-level categorical variable (<9 years, <high school, high school degree/GED, some college/associate’s degree, bachelor’s degree or more). I convert data from earlier waves into these five categories, using years of completed schooling. Since state-level compulsory schooling laws in the United States mandate schooling until at least age 16 (National Center for Education Statistics 2015), over one-third (36%) of the sampled population with less than nine years of schooling is foreign-born. To minimize bias from nativity, I combine categories <9 years and <high school into one category, <high school.

#### 2.2.3 Measures of adiposity

In the main analysis, I examine two measures of BMI: BMI at the time of survey and lifetime maximum BMI. BMI, calculated as weight in kilograms over height in meters squared, is a commonly used estimator of adiposity. Values between 18.5 kg/m^2^ and 25 kg/m^2^ are considered healthy, values between 25 kg/m^2^ and 30 kg/m^2^ are considered overweight, and values of 30 kg/m^2^ or higher are considered obese. I use the terms adiposity and obesity interchangeably throughout this study.

I construct a continuous measure of BMI at survey time using height and weight, both measured at a mobile examination center at the time of examination. Consistent with previous studies, I construct a continuous variable for maximum BMI using measured height and self-reported highest-ever weight. Preston, Fishman, and Stokes (2015) show that bias from weight misreporting in estimates of the mortality consequences of obesity is greatly reduced if using a continuous measure of BMI.

In a sensitivity analysis, I examine two additional estimates of adiposity. The first is BMI at age 25, constructed using height measured at the time of survey and self-reported weight at age 25. The second is waist circumference in centimeters, measured at the time of survey.

#### 2.2.4 Other covariates

Age, sex, and race/ethnicity are correlated with BMI (Elo, Mehta, and Preston 2017; Heymsfield et al. 2016; Reynolds, Satio, and Crimmins 2005; Zhang and Wang 2004) and included as additional covariates, as are continuous variables for age at baseline and years since interview.

I also include a categorical variable for smoking history, capturing never-smokers, former smokers, and current smokers. Because smokers, especially life-long smokers, have a higher risk of death and are more likely to have a healthy BMI (Audrain-McGovern and Benowitz 2011; Stokes and Preston 2016a), cigarette use can obscure the relationship between obesity and mortality (Stokes and Preston 2016a). Threats of confounding are particularly serious in studies of educational attainment and mortality, given the inverse relationship between smoking and education (Ahmed et al. 2015).

### 2.3 Analytic strategy

I model the relationship between education and mortality using discrete-time logistic regressions. The full models include variables for age at baseline, years since interview, sex, race/ethnicity, education, smoking, and one to two adiposity measures. I compare models using model performance criteria Akaike information criterion (AIC) and Bayesian information criterion (BIC).

Next, I estimate the percentage of the education-mortality relationship that is mediated by educational differences in adiposity. In a logistic model, coefficients can change across nested models both because an added variable *z* (adiposity) mediates the relationship between the independent variable of interest *x* (education) and the dependent variable *y* (mortality) and because the underlying scale of the model has shifted (Karlson, Holm, and Breen 2012). It is not sufficient, therefore, to estimate the percentage of the education-mortality relationship that is associated with obesity by examining the percent change in the education coefficients across nested models before and after controlling for adiposity. If done this way, it is unclear how much the coefficients for education have changed due to (1) obesity mediating the association between education and mortality and (2) the shift in the scale caused by the introduction of a new variable. Failure to correct for rescaling often underestimates the mediating role of variable *z* in the relationship between *x* and *y*, “increasing the likelihood of our concluding, incorrectly, that changing *z* would have little or no impact on the *x-y* relationship” (Karlson, Holm, and Breen 2012: 288).

To address this issue I apply a method proposed by Karlson, Holm, and Breen (2012) (KHB), using the *khb* command in Stata version 15 (StataCorp 2017). To isolate the separate effects of rescaling and confounding, the method linearly regresses *z* (adiposity) on *x* (education) and uses the *x*-residualized *z*-variable in the full model to remove any correlation between *x* and *z*. Because the original and corrected models have the same scale, we can determine the change in the coefficient attributable to confounding from variable *z*, net of rescaling, by subtracting the coefficient for *x* in the original model with the *x*-*z* correlation from the coefficient for *x* in the corrected model without the correlation. The underlying discrete-time logistic regressions model both smoking and adiposity as mediators in the relationship between education and mortality. The models additionally include demographic controls for age at baseline, years since interview, sex, and race/ethnicity. In a sensitivity analysis, I repeat the *khb* decomposition examining the contribution of two additional estimates of adiposity to educational mortality differences: waist circumference measured at survey time and BMI at age 25.

Finally, I repeat the above *khb* procedure restricting the sample by sex and nonsmoking status using only the best performing estimate of adiposity. Since smoking increases the risk of death and is inversely related to obesity (Audrain-McGovern and Benowitz 2011; Stokes and Preston 2016a), measurement error in smoking status, such as the inability to account for smoking duration and intensity, may bias estimates of the relationship between obesity and mortality (Stokes and Preston 2016a). Although not generalizable to the overall population, results for never-smokers present a less biased picture of obesity’s contribution to mortality differences.

## 3. Results

### 3.1 Descriptive statistics and age-standardized mortality rates

Table 1 presents characteristics of the sample by educational attainment, weighted to be nationally representative of the noninstitutionalized population. The majority (61%) of respondents have at most a high school degree, and just over one-fifth (21%) of respondents have some college experience or an associate’s degree, but no bachelor’s degree. Respondents with bachelor’s degrees are more likely than those without four-year degrees to be younger non-Hispanic white never-smokers. At survey time one-quarter (25%) of college-educated respondents were obese, compared to one-third (35%) of those with a high school degree. Roughly one-third (36%) of college graduates reported having ever been obese, as measured by maximum BMI, compared to nearly half (49%) of those with a high school degree. The relationship between education and past obesity is somewhat more linear than between education and obesity at survey time, suggesting that BMI at survey time masks heterogeneity in weight histories. Some of this heterogeneity is illustrated by the negative association between education and weight loss shown in Table 1. On average, those with less than a high school degree weighed 2.3 kg/m^2^ less at survey time than they did at their highest weight, compared to college graduates who lost 1.6 kg/m^2^. Differences in weight loss may be influenced both by illness and by the educational patterning of current smoking (Ahmed et al. 2015), as smoking is negatively correlated with current weight (Audrain-McGovern and Benowitz 2011). Among those with less than a high school degree, 32% reported being current smokers, compared to 11% of those with at least four years of college.

Table 1 also shows mortality rates by education and sex for the studied population, calculated using weighted deaths and person-years, and adjusted using the age distribution of adults aged 40–84 in the 2000 Census. As expected, education and mortality are highly negatively correlated. Those without a high school degree were more than twice as likely as college-educated respondents to die during follow-up (12.41 deaths vs. 5.58 deaths per 1,000 for females and 16.61 vs. 7.10 deaths per 1,000 for males, respectively). Using the same age standard, the death rate for the overall population of 40–84-year-olds in 2000 National Vital Statistics data is 11.33 for females and 17.35 for males (Miniño et al. 2002), compared to 10.85 for females and 14.26 for males in NHANES. Mortality is slightly lower in the sample both because NHANES, unlike Vital Statistics, excludes individuals who are institutionalized at baseline and because I additionally exclude those who are underweight at baseline.

Table 2 presents characteristics of the sample for never and current smokers by sex. Current smokers are less likely than never-smokers to have a bachelor’s degree (11% vs. 26%, respectively, for females and 13% vs. 40% for men). Current smokers are also less likely to be currently obese, though these differences diminish if we consider having ever been obese. Although never-smoker females were 23% more likely than currently smoking females to be obese at the time of survey, they were only 8% more likely to have ever been obese. For males, these figures are 36% and 7%, respectively. Despite current smokers’ healthier weight, current smokers experienced more than twice the mortality rates of never-smokers, illustrating smoking’s confounding role in the relationship between obesity and mortality. The age-standardized mortality rate for currently smoking males is 23.10 annual deaths per 1,000, about three times the rate of 7.82 deaths per 1,000 among never-smoker males. For females, these rates are 16.27 and 6.16 deaths per 1,000, respectively.

### 3.2 Model performance

Table 3 presents odds ratios of dying from all causes during the follow-up period. Model 1 estimates the baseline relationship between education and mortality, controlling for age, sex, race/ethnicity, and smoking. In order to highlight differences between the extreme ends of the education distribution, having a bachelor’s degree serves as the reference category. Models 2 and 3 each add a different estimate of adiposity: survey time BMI and maximum BMI, respectively. Model 4 adds both adiposity estimates simultaneously, and Model 5 includes both maximum BMI and BMI units lost from maximum weight at survey time. Although previous research has shown the inclusion of a quadratic term for obesity estimates to improve model performance (Kivimaki et al. 2008), this was only the case for survey time BMI and is therefore not shown for maximum BMI. I did not find evidence of significant interactions between adiposity+sex, adiposity+education, education+sex, or education+smoking in any of the models. There is evidence of an interaction between age and adiposity throughout. However, since the focus of the main analysis is the change in age-adjusted mortality differences by education once controlling for adiposity, an interaction between age and adiposity does not affect the main findings. To avoid including several age+adiposity interactions in Model 4, and since these interactions had only a modest effect on the odds ratios in Table 3, age interactions are not shown in this table.

In Model 1 those with less than a high school degree are more than twice as likely as college graduates to die during follow-up (odds ratio of 2.015), even after controlling for differences in age, sex, race/ethnicity, and cigarette smoking. Those with a high school degree or some college are also significantly more likely to die than those with a four-year degree (odds ratios of 1.651 and 1.431, respectively). If we account for the fact that less educated people are more likely to be obese at the time of survey in Model 2, the survival advantage of having a college degree is somewhat lessened. Here the odds ratio of dying for those without a high school degree decreases from 2.015 in Model 1 to 1.941 in Model 2. The odds ratio of dying for this group declines further to 1.820 in Model 3, when controlling for the fact that members of this group are more likely to have ever been obese. There are similar declines in the odds ratios across Models 1, 2, and 3 for high school graduates and those with some college. I account for rescaling using Karlson, Holm, and Breen (2012) decompositions in Section 3.3 below.

Model 4 controls for maximum and survey time BMI simultaneously. The odds ratios for education in Model 4 are somewhat larger than those in the maximum BMI model and somewhat smaller than those in the survey time BMI model (though the differences are not statistically significant). This indicates that, when analyzed jointly in the context of educational mortality disparities, survey time and maximum BMI may work in opposite directions. As shown in Table 1, less educated individuals are both more likely to have ever weighed more and to have lost more weight (likely due to illness). This reality is captured when controlling for both survey time and maximum BMI in a model of mortality. The presence of maximum BMI in Model 4 shrinks mortality disparities by accounting for having ever been obese, while survey time BMI widens disparities by capturing weight loss.

That Model 4 is detecting illness-induced weight loss is further evidenced by the odds ratios for the adiposity measures themselves. The nadir of the relationship between survey BMI and mortality in Model 2 is 18.7 kg/m^2^, meaning that a higher BMI at the time of survey is associated with increased mortality above 18.7 kg/m^2^. In Model 4, however, survey time BMI does not have a positive association with mortality until a BMI value of 72 kg/m^2^. The odds of dying in Model 4 are higher for obese individuals who have lost weight than for similarly obese individuals who have maintained their weight: a finding indicative of reverse causation.

Model 5, which controls for both maximum BMI and weight lost from maximum BMI, further supports the claim that reverse causation is at work in Model 4. In Model 5 the odds of dying increase by 3.5% for each additional unit of maximum BMI and decrease by 5% for each BMI unit of weight loss. In this model the survival advantage of a college degree shrinks to an all-time low: we can explain a greater share of the mortality gradient by controlling for the fact that while people with lower levels of education tend to have weighed more than their higher educated counterparts in the past, they also tend to have lost more weight, presumably because of illness. However, the goal of the present analysis is not to account for the largest share of the gradient possible, but rather to estimate the share of the gradient attributable to differential adiposity – not adiposity and weight loss. Models 4 and 5 illustrate that when both maximum and current BMI are in the model, the mortality hazards associated with weight loss become a prominent factor. This reverse causal path is not the subject of this paper and creates a statistical disturbance in investigating the subject of interest. As a result, I discard Models 4 and 5 for purposes of answering the fundamental question posed in this paper.

Having thus excluded Models 4 and 5, I turn to model performance criteria AIC and BIC, presented in Table 3. Model 3, using maximum BMI, performs better than Models 1 and 2 (AIC values of 35,320 versus 35,461 and 35,417, respectively; BIC values of 35,456 versus 35,587 and 35,563, respectively). This finding is consistent with previous research using model selection criteria to compare models using maximum and survey time BMI (Stokes and Preston 2016b).

### 3.3 Karlson, Holm, and Breen decompositions

**Figure 1:**
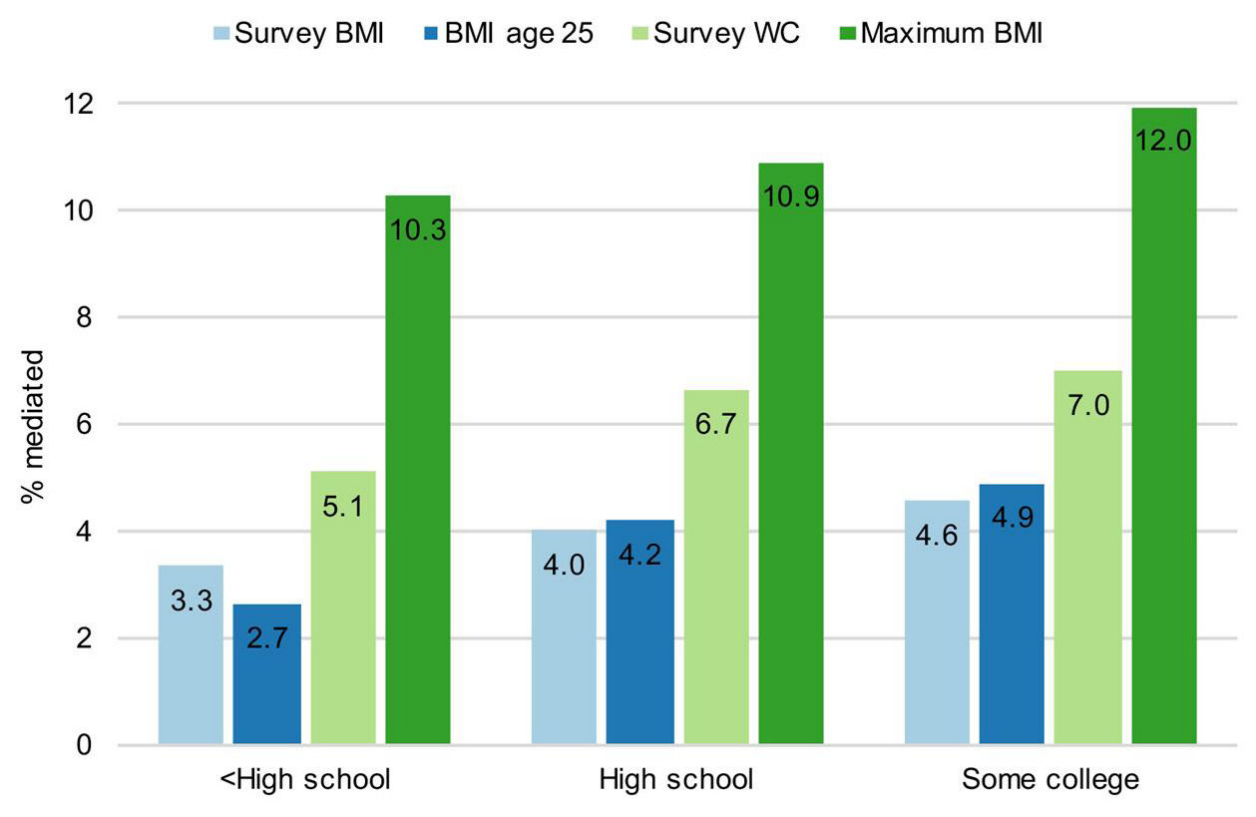
Percentage of mortality differences between college graduates and others mediated by adiposity, by adiposity measure *Notes*: Karlson, Holm, and Breen (2012) decompositions of discrete-time logistic regressions. All models include adiposity and smoking status as mediators of the relationship between education and mortality. All models include additional covariates for age at baseline, years since interview, sex, and race/ethnicity. Survey WC: waist circumference measured at the time of survey. *Source*: NHANES 1988–2011.

Figure 1 displays direct results of the KHB decompositions, estimating the percentage of the college-graduate mortality advantage that is associated with differences in adiposity, using four different measures of adiposity. When estimated using maximum BMI, the best performing variable, adiposity is associated with between 10.3% and 12% of differences between those with and without four-year degrees. This is roughly three times the explanatory power of survey time BMI, which is associated with between 3.3% and 4.6% of differences. Figure 1 also includes results examining the explanatory power of two additional measures of body fat: waist circumference at the time of survey and BMI at age 25. BMI at age 25 performs similarly to survey time BMI, explaining 2.7–4.9% of the differential, while waist circumference is associated with a somewhat larger proportion of mortality differences (5.1–7%).

**Figure 2:**
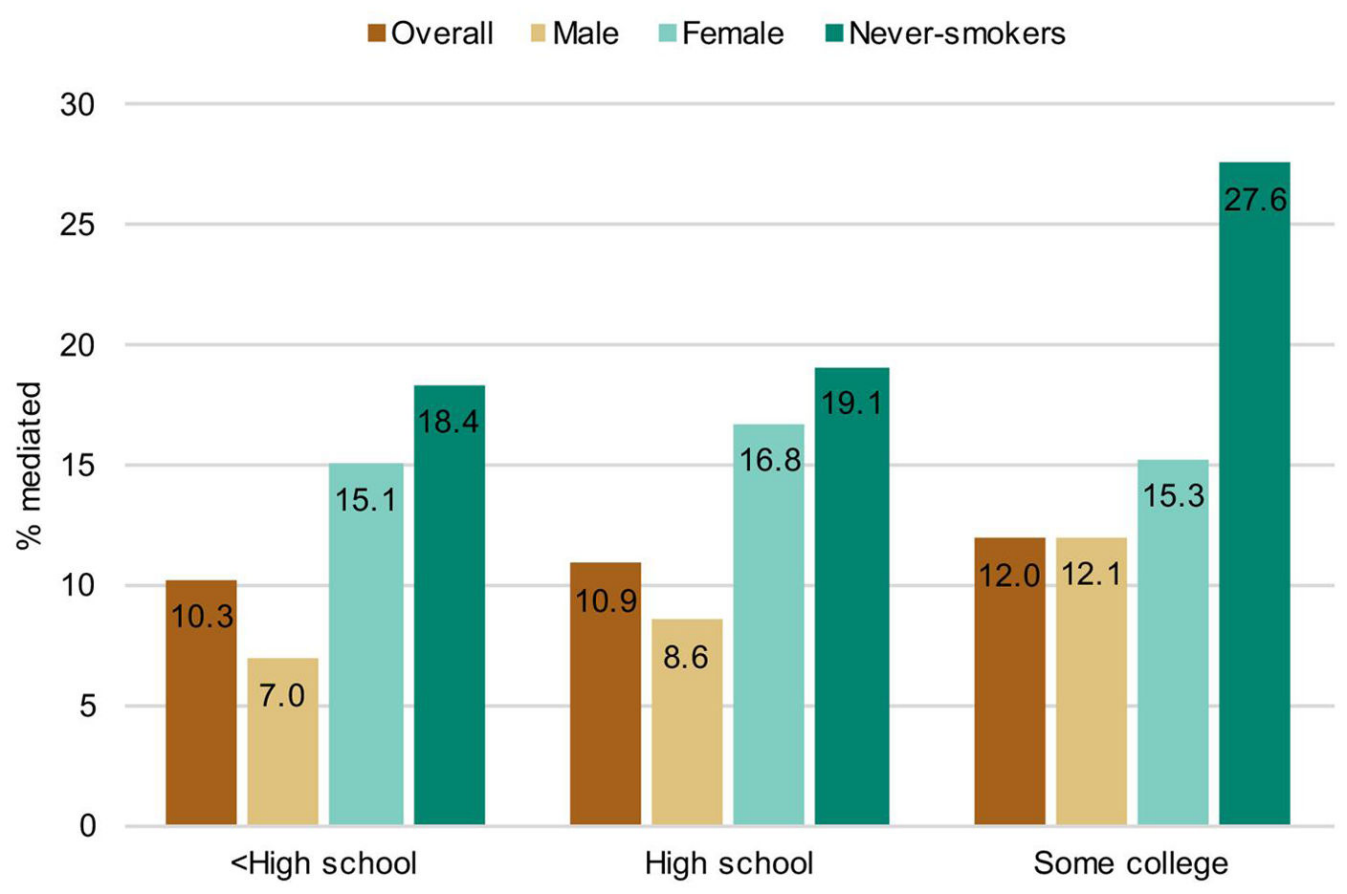
Percentage of mortality differences between college graduates and others mediated by maximum BMI, by sex and smoking status *Notes*: Karlson, Holm, and Breen (2012) decompositions of discrete-time logistic regressions. All models include adiposity and smoking status (if applicable) as mediators of the relationship between education and mortality. All models include additional covariates for age at baseline, years since interview, sex (if applicable), and race/ethnicity. *Source*: NHANES 1988–2011.

Figure 2 presents results by sex and smoking status, using only maximum BMI. Among women, differences in maximum BMI are associated with 15.1–16.8% of the mortality advantage of college graduates. For men, this proportion is smaller and more variable, ranging from 7–12.1%, likely due in part to the higher prevalence of smoking among men (Ahmed et al. 2015) and the stronger education-obesity gradient among women (Yu 2012). Among never-smokers, adiposity is an even more powerful mediator in the relationship between education and mortality: between 18.4% and 27.6% of the mortality advantage for college graduates among never-smokers is associated with differences in maximum BMI.

## 4. Discussion and conclusion

Despite large gains in life expectancy in the United States and other high-income countries over the past century, substantial differences in mortality conditions persist across subpopulations. One of many stratifying dimensions is educational attainment: people with fewer years of schooling live fewer years, and spend fewer of these years healthy (see, e.g., Elo 2009; Hayward, Hummer, and Sasson 2015; Laditka and Laditka 2016; Mackenbach et al. 2008; Mackenbach et al. 2015). Using nationally representative data, I find that educational differences in adiposity contribute to this disparity in the United States, though the size of adiposity’s contribution is sensitive to how adiposity is measured.

The majority of prior work, finding little or no association between obesity and educational differences in mortality, relies exclusively on a single cross-sectional observation of BMI at the time of survey. This approach can underestimate the risks of obesity since reverse causation due to illness biases the mortality risks of obesity downward (Stokes and Preston 2016a, 2016b). Given that less educated people are more likely to contract illnesses that induce weight loss, the likelihood of reverse causation is especially great among less educated people, making analyses of socioeconomic differences in mortality particularly sensitive to the biases accompanying BMI at the time of survey. A growing body of research documents that lifetime maximum BMI is a more reliable indicator of adiposity, both because it skirts issues of reverse causation and because it contains information relating to weight history, which may have enduring predictive power for mortality (Stokes and Preston 2016a). This paper is the first to exploit the advantages of maximum BMI to examine socioeconomic disparities in mortality.

The main analysis compared models of mortality using BMI at the time of survey and lifetime maximum BMI, finding that, based on model performance criteria AIC and BIC, a model with maximum BMI best explained the observed data. This result is consistent with existing research demonstrating the strengths of maximum BMI as a variable to estimate obesity’s relationship with mortality (Preston et al. 2015, 2016a). I find that having ever been obese is associated with increased mortality, net of current weight, and that ever being obese accounts for roughly three times more of the educational gradient in mortality than does current weight status. Between 10.3% and 12% of the mortality advantage of college graduates over nongraduates is associated with differences in maximum BMI, compared to between 3.3% and 4.6% with survey time BMI. One explanation for the greater explanatory ability of maximum BMI is that less educated individuals are more likely to contract illnesses that result in weight loss: a distinction overlooked by survey time BMI. A second is that maximum BMI captures elements of weight history predictive of mortality (Preston, Mehta, and Stokes 2013), elements which are likely to vary with SES (Pampel, Krueger, and Denney 2010).

A limitation of this study is that the maximum BMI measure does not indicate at what age an individual reached peak weight, nor for how long that weight was maintained. A second limitation of this analysis is that maximum weight in NHANES is based on recalled highest-ever weight and, judging from patterns observed in the Health and Retirement Survey (HRS), likely overestimated. Nevertheless, measured and recalled peak weight in the HRS are highly correlated at 0.948 (Stokes and Ni 2016). Using a continuous measure of BMI, rather than a categorical one, reduces much of the bias from weight self-reporting (Preston, Fishman, and Stokes 2015). A third limitation is that the demonstrated relationship is not causal: having ever been obese is associated with disparities in mortality. Examining the pathways between maximum BMI and mortality, and whether they are causal, are important avenues for future research. This study also does not provide insight into the upstream factors shaping differential obesity prevalence by educational attainment in the first place.

The superior performance of maximum BMI highlights the need for health surveys to collect data on maximum weight. Without declines in obesity prevalence, the role of obesity in shaping the education-mortality relationship is likely to grow. Although the sharp decline in smoking in recent years (Ahmed et al. 2015) is a positive development for health outcomes, it also indicates that obesity may take on a larger role in driving mortality differentials. Among the growing number of never-smokers, maximum BMI is associated with over one-fifth (18.4–27.6%) of the survival advantage of college graduates, underscoring the urgency of leveling differences in obesity prevalence by educational attainment.

## Figures and Tables

**Table 1:** Sample characteristics of adults aged 40–74 at baseline by educational attainment, NHANES 1988–2011^a^

Characteristics	<High school (95% CI)	High school (95% CI)	Some college, associate’s degree (95% CI)	Bachelor’s degree or more (95% CI)	Total (95% CI)
**Adjusted mortality rate (per 1,000)**^b^					
Female	12.41	8.44	7.15	5.58	10.85
Male	16.61	15.18	11.72	7.10	14.26
**Adiposity**					
Currently obese^c^ (%)	36.6 (34.8 to 38.7)	34.6 (32.7 to 36.5)	34.8 (32.9 to 36.7)	25.0 (23.3 to 26.7)	32.7 (31.5 to 33.8)
Ever obese^c^ (%)	52.9 (51.3 to 54.4)	49.4 (47.3 to 51.5)	48.7 (46.5 to 50.8)	35.7 (33.5 to 37.9)	46.5 (45.2 to 47.8)
BMI survey (mean)	28.9 (28.7 to 29.1)	28.7 (28.5 to 29.0)	28.7 (28.4 to 29.0)	27.4 (27.2 to 27.7)	28.4 (28.3 to 28.6)
BMI max (mean)	31.5 (41.3 to 31.7)	30.9 (30.7 to 31.2)	30.8 (30.5 to 31.1)	29.2 (28.9 to 29.5)	30.6 (30.4 to 30.8)
BMI lost^c^ (mean)	2.3 (2.2 to 2.4)	2.0 (1.8 to 2.1)	1.9 (1.8 to 2.0)	1.6 (1.5 to 1.7)	1.9 (1.9 to 2.0)
**Age (mean)**					
Age at survey	57.2 (56.8 to 57.6)	54.9 (54.4 to 55.4)	53.3 (53.0 to 53.7)	53.0 (52.6 to 53.4)	54.5 (54.3 to 54.8)
Age at follow-up	68.4 (67.9 to 69.0)	66.5 (65.9 to 67.1)	63.3 (62.7 to 63.7)	63.9 (63.3 to 64.5)	65.5 (65.1 to 65.9)
**Male (%)**	49.2 (47.7 to 50.8)	44.5 (43.1 to 45.9)	46.4 (44.1 to 48.6)	54.8 (53.1 to 56.4)	48.6 (47.9 to 49.3)
**Race/ethnicity (%)**					
Non-Hisp. white	58.4 (54.4 to 62.4)	81.2 (79.2 to 83.3)	79.5 (77.5 to 81.5)	85.6 (83.6 to 87.6)	76.9 (75.0 to 78.9)
Non-Hisp. black	15.9 (14.1 to 17.8)	9.9 (8.7 to 11.1)	9.9 (8.7 to 1.2)	5.6 (4.8 to 6.4)	10.1 (9.2 to 11.1)
Hispanic	21.0 (17.9 to 24.1)	5.9 (4.9 to 6.9)	6.7 (5.6 to 7.7)	3.5 (2.8 to 4.3)	8.8 (7.6 to 10.0)
Other	4.6 (3.4 to 5.9)	2.9 (2.2 to 3.7)	3.9 (3.2 to 4.7)	5.3 (3.7 to 6.9)	4.1 (3.4 to 4.8)
**Smoking status (%)**					
Never	37.7 (35.6 to 39.7)	41.0 (39.1 to 42.8)	44.3 (42.4 to 46.1)	55.0 (52.9 to 57.2)	44.6 (43.3 to 45.8)
Former	30.8 (29.3 to 32.2)	31.5 (29.8 to 33.1)	34.0 (31.9 to 36.0)	34.2 (32.1 to 36.2)	32.6 (31.5 to 33.6)
Current	31.6 (30.0 to 33.2)	27.6 (26.1 to 29.0)	21.8 (20.0 to 23.6)	10.8 (9.4 to 12.2)	22.8 (21.8 to 23.8)

N	7,987	5,777	4,878	4,061	22,703
Deaths	1,971	968	521	324	3,784
Person-years	79,185	58,328	40,781	36,772	215,066

*Notes*:

a Results reflect sample weighting except N, deaths, and person-years.

b Mortality rates include mortality until age 85 at follow-up. Rates are age-adjusted using the age distribution of 40–84-year-olds in the 2000 Census.

c ‘Currently obese’ estimated as BMI at survey time greater than 30. ‘Ever obese’ estimated as maximum BMI greater than 30. ‘BMI lost’ estimated as max BMI – survey BMI.

**Table 2:** Sample characteristics of adults aged 40–74 at baseline, by sex and smoking status, NHANES 1988–2011^a^

Characteristics	Female (95% CI)		Male (95% CI)	
	Never-smoker	Current smoker	Never-smoker	Current smoker
**Adjusted mortality rate(per 1,000)**^b^	6.16	16.27	7.82	23.10
**Adiposity**				
Currently obese^c^ (%)	36.4 (34.7 to 38.1)	29.7 (27.7 to 31.6)	32.3 (29.7 to 35.0)	23.7 (21.5 to 25.9)
Ever obese^c^ (%)	45.8 (44.0 to 47.6)	42.5 (40.2 to 44.8)	47.0 (44.4 to 49.7)	44.1 (41.6 to 46.6)
BMI survey (mean)	28.8 (28.6 to 29.0)	27.5 (27.2 to 27.9)	28.7 (28.5 to 29.0)	27.1 (26.9 to 27.3)
BMI maximum (mean)	30.6 (30.3 to 30.8)	30.6 (29.8 to 30.5)	30.7 (30.4 to 31.0)	30.1 (29.8 to 30.3)
BMI lost^c^ (mean)	1.4 (1.3 to 1.5)	2.3 (2.2 to 2.5)	1.8 (1.7 to 2.0)	2.9 (2.7 to 3.0)
**Age (mean)**				
Age at survey	54.8 (54.4 to 55.3)	52.7 (52.2 to 53.1)	52.9 (52.4 to 53.3)	52.0 (51.6 to 52.4)
Age at follow-up	65.9 (65.4 to 66.5)	63.7 (63.0 to 64.4)	62.9 (62.3 to 63.5)	63.1 (62.5 to 63.6)
**Race/ethnicity (%)**				
Non-Hisp. white	71.8 (69.1 to 74.5)	76.4 (73.5 to 79.3)	76.7 (74.4 to 79.1)	72.7 (70.0 to 75.5)
Non-Hisp. black	11.4 (10.1 to 12.7)	13.3 (11.5 to 15.0)	9.1 (8.1 to 10.1)	14.2 (12.6 to 15.8)
Hispanic	11.0 (9.5 to 12.6)	7.0 (5.4 to 8.5)	9.4 (8.1 to 10.7)	8.7 (6.8 to 10.5)
Other	5.9 (4.5 to 7.2)	3.42 (2.2 to 4.6)	4.8 (3.5 to 6.1)	4.4 (3.2 to 5.6)
**Education (%)**				
<High school	20.7 (19.0 to 22.3)	29.2 (26.8 to 31.5)	14.9 (13.4 to 16.4)	31.1 (28.2 to 34.1)
High school	29.7 (28.0 to 31.4)	36.9 (34.4 to 39.5)	21.6 (19.8 to 23.4)	33.5 (30.9 to 36.1)
Some college	24.0 (22.3 to 25.8)	22.9 (20.5 to 25.2)	23.2 (21.2 to 25.2)	22.7 (20.5 to 24.9)
Bachelor’s degree	25.6 (23.5 to 27.7)	11.0 (9.2 to 12.9)	40.3 (37.8 to 42.8)	12.7 (10.5 to 14.9)

N	6,550	2,192	3,831	3,033
Deaths	727	448	431	792
Person-years	64,662	21,204	33,699	28,534

*Notes*:

a Results reflect sample weighting except N, deaths, and person-years.

b Mortality rates include mortality until age 85 at follow-up. Rates are age-adjusted using the age distribution of 40–84-year-olds in the 2000 Census.

c ‘Currently obese’ estimated as BMI at survey greater than 30. ‘Ever obese’ estimated as maximum BMI greater than 30. ‘BMI lost’ estimated as max BMI – survey BMI.

**Table 3:** Odds ratios of dying from all-cause mortality for adults aged 40–74 at baseline, NHANES 1988–2011^a^

Characteristics	Model 1: Baseline	Model 2: Survey BMI	Model 3: Max BMI	Model 4: Max + survey BMI	Model 5: Max – survey BMI
**Education (ref: BA or more)**					
<High school	2.015 (1.646 to 2.468)	1.941 (1.585 to 2.375)	1.820 (1.491 to 2.221)	1.822 (1.494 to 2.222)	1.804 (1.480 to 2.200)
High school	1.651 (1.383 to 1.970)	1.608 (1.346 to 1.921)	1.533 (1.285 to 1.828)	1.553 (1.300 to 1.854)	1.536 (1.287 to 1.832)
Some college	1.431 (1.177 to 1.740)	1.404 (1.156 to 1.707)	1.354 (1.115 to 1.644)	1.356 (1.121 to 1.641)	1.347 (1.112 to 1.632)
**Age at baseline**	1.102 (1.094 to 1.110)	1.103 (1.095 to 1.112)	1.104 (1.095 to 1.112)	1.103 (1.095 to 1.111)	1.102 (1.094 to 1.110)
**Years since interview**	1.109 (1.098 to 1.120)	1.111 (1.100 to 1.123)	1.114 (1.103 to 1.125)	1.115 (1.104 to 1.127)	1.115 (1.103 to 1.126)
**Sex (ref: female)**	1.352 (1.236 to 1.479)	1.405 (1.274 to 1.550)	1.361 (1.240 to 1.495)	1.363 (1.237 to 1.503)	1.325 (1.208 to 1.453)
**Race (ref: NH white)**					
NH black	1.388 (1.248 to 1.545)	1.326 (1.190 to 1.477)	1.289 (1.156 to 1.438)	1.288 (1.155 to 1.436)	1.295 (1.160 to 1.445)
Hispanic	0.888 (0.762 to 1.035)	0.896 (0.771 to 1.040)	0.894 (0.774 to 1.032)	0.901 (0.779 to 1.042)	0.894 (0.774 to 1.032)
Other	1.153 (0.827 to 1.608)	1.185 (0.845 to 1.660)	1.246 (0.872 to 1.779)	1.220 (0.851 to 1.748)	1.240 (0.862 to 1.783)
**Smoker (ref: never)**					
Former	1.470 (1.312 to 1.647)	1.473 (1.319 to 1.646)	1.466 (1.310 to 1.641)	1.476 (1.310 to 1.663)	1.467 (1.302 to 1.654)
Current	2.693 (2.365 to 3.066)	2.818 (2.467 to 3.219)	2.891 (2.538 to 3.293)	2.690 (2.355 to 3.074)	2.737 (2.397 to 3.125)
**Adiposity**					
BMI at survey		0.928 (0.889 to 0.968)		0.866 (0.827 to 0.907)	
BMI at survey (squared)		1.002 (1.001 to 1.002)		1.001 (1.001 to 1.002)	
BMI maximum			1.046 (1.038 to 1.054)	1.091 (1.078 to 1.105)	1.035 (1.026 to 1.044)
BMI lost					1.050 (1.038 to 1.063)
**Constant**	0.000 (0.000 to 0.000)	0.000 (0.000 to 0.000)	0.000 (0.000 to 0.000)	0.000 (0.000 to 0.000)	0.000 (0.000 to 0.000)

AIC	35,461.47	35,417.03	35,320.40	35,098.85	35,146.26
BIC	35,586.50	35,562.89	35,455.84	35,255.14	35,292.13
N			22,703		
Deaths			3,729		
Person-years			215,066		

*Notes*:

a Results reflect sample weighting except N, deaths, and person-years. 95% confidence intervals are shown.
